# Monthly Summary

**Published:** 1892-11

**Authors:** 


### Monthly Summary.

---

VULCANIZING.—Dr. Geo. B. Snow says in a recent letter:—"I find black rubber a rather treacherous material to work by the directions I have given. I have had what for lack of a better name I shall call 'blow holes,' when I am certain the case was well packed. I finally hit upon the plan of not fully closing the flask—interposing strips of thin metal of a thickness I thought sufficient to compensate for shrinkage. As the rubber expands in heating to 320, some of it is forced into the gateways, and a constant pressure is thus exerted upon the contents of the flask until vulcanization and its consequent shrinkage begins. At the proper time, the vulcanizer is opened and the pieces withdrawn, and the case re-heated to allow the flask to close. This puts the rubber into the same conditions as in the usual practice, until after vulcanization is nearly completed."

A pamphlet on the "new process" of vulcanizing, embodying the above in working detail, may be looked for in the near future.—*Odontographic Journal.*

---

GUM LANCING AN IMPORTANT THERAPEUTICAL MEASURE.—Clinically, I am absolutely sure that I have seen convulsions, sick stomach, great restlessness, fever, and various other functional disturbances in young children, immediately cured by the use of the gum lancet, after the failure of various other well directed measures for relief. Theoretically, I am in accord with Dr. Kirk, in believing that Dr. Forchheimer absolutely misses the point of the matter, by his failure to understand that the good achieved is not due to the local blood letting or to the relief of the inflammation of the gum, but to the removal of the backward pressure upon an extradordinary sensitive and, at such times, congested tooth pulp. As was long ago pointed out by Dr. J. W. White, at the period of eruption the roots of the teeth are incomplete.

"Instead of the conical termination and minute foramen, which characterize a perfected tooth, the aperture is nearly as large as the tooth itself, and thus when the sensitive pulp, composed of connective tissue, blood vessels, and nerves, is in a condition of irritation because of the morbid activity of the process of dentition—augmented vascular and nervous action—there may be produced a hyperemia sufficient, possibly, to cause the protrusion of a part of the mass from the incomplete aperture of the root, giving abundant cause for extreme constitutional disturbance."

I have myself seen a seemingly incurable epilepsy in an adult permanently cured by the removal of a persistent milk or first dentine tooth. Amaurosis and various other conditions in the adult, are well known to be the result of irritation of the trigeminal nerve by faulty teeth. How much more evil is to be expected from teeth irritation in the child!

In conclusion, I reaffirm that whatever the theory in the matter may be, I am positive that gum lancing is the most important therapeutic measure. It is essential, however, that it should be thorough, and with the object of dividing the dense tissues that bind down the teeth.—WOOD (H. C.), *University Medical Magazine.*

---

NEW METHOD OF ARTIFICIAL RESPIRATION.—M. Raborde reported two cases of apparent drowing in which he had excited the respiratory action by employing forcible rhythmic traction upon the tongue, backwards and forwards. A spoon or the finger is used to keep the mouth open and fix the base of the tongue. At the same time the thorax is enveloped in cloths wrung out of hot water, care being taken not to scald it. This idea was suggested to the author by laboratory experiments upon animals asphyxiated by chloroform, he having noticed that when the tongue was pulled out to straighten the air-passages before commencing artificial respiration, respiratory efforts were frequently excited. This led him to investigate, with the result that he discovered this method which he believes is superior to all others. "In all cases—and this is my conclusion—never despair, no matter how probable death may appear," is the advice with which he closes the communication which was made to the Academie de Medecine de Paris.—*Le Bulletin Medical*, July 6, '92.

---

SIMPLE TESTS FOR IMPURITIES IN WATER.—The following methods of determining the presence of impurities in water are given by Walling: 1. For organic matter put a little of the sample into a beaker, add two or three drops of dilute sulphuric acid and color distinctly with a solution of permanganate of potassium. If much organic matter is present the color of permanganate becomes discharged almost immediately; if less or very little, it takes longer to decolorize. If the color has not changed in twenty-five or thirty minutes it is safe to assume that organic matter was not present. This is a tolerably test 2. For nitrites, a little sulphuric acid added to the water forms nitrous acid if nitrites are present, which is easily detected by its power of liberating iodine from iodide of potassium. A little starch paste is mixed with a small quantity of a solution of potassium iodide, and the mixture added to the suspected water containing the sulphuric acid. If nitrites are present the nitrous acid formed liberates the iodine from the iodide, which turns blue with starch. This indirect method is a ready means for detecting the nitrites if present in not too small a quantity. 3. Nitrates are detected by converting into nitric acid, which turns morphia red. A portion of water is evaporated to dryness, the residue treated with a drop of strong sulphuric acid (which makes nitric acid of the nitrate) and a portion of morphine added. If nitrate is present the morphine gives red color. 4. For ammonia, Nessler's reagent is by far the best test. It may be made by dissolving eighteen grains of oxide of potassium in a little water, adding solution of mercuric chloride until the red iodide of mercury first formed redissolves upon agitation. To this is added a solution of fifty grains caustic potassa and distilled water to make eight ounces. This reagent will detect

0.00375 of a grain in a pint of water by giving a yellow color. A reddish color or precipitate forms with larger quantities of ammonia. 5. Albuminoid matter requires a more elaborated proceeding for its detection. If all of the above were found it is hardly necessary to go to the trouble of looking for albuminoids; the water would be unwholesome even if they were not present.—*Pharmaceutical Era.*

---

COCAINE.—M. Bignon (*Bull. de Therap.*) formulates the results of his experimental researches as follows: (1) Cocaine parts with its valuable anæsthetic properties in a decidedly acid solution. (2) In such solutions its power as an anæsthetic is not destroyed, but becomes latent. (3) The anæsthetic property is fully restored by simply neutralizing the acid. (4) All the mineral and organic acids used in the experiments operated in the above manner. (5) The anæsthetic energy of cocaine attains its maximum when, all its acid having been neutralized, the alkaloid is held in suspension by a slightly alkaline liquid. This preparation the author calls milk of cocaine. (6) Milk of cocaine is obtained by precipitating the hydrochlorate or other salt of cocaine by a slight excess of carbonate of soda. The bicarbonate does not act so well. (7) Most of the cocaine salts, especially the hydrochlorate, retain an appreciable quantity of acid; hence their solutions do not possess the full anæsthetic potency of the alkaloid; i. e., a portion of the same remains latent. (8) The anæsthetic power of cocaine salts varies greatly in different cases. Some crystallized hydrochlorates, though perfectly pure, are so acid that 10 centigrams, simply dissolved in water, are no more efficacious than half the quantity when neutralized and made into milk of cocaine. (9) The surgeon's first care, therefore, before using a solution of any cocaine salt, should be to assure himself of its neutrality; this can only be obtained at a sacrifice of limpidity; it is accompanied by some degree of opalescence (10) The difference in the quantities required for production of anæsthesia is in most cases explainable by the difference in the acidity of the solutions employed.

(11) The effect of subcutaneous injections of milk of cocaine on the human subject has yet to be ascertained. Experiments on dogs have given encouraging results in this direction, but the author has not succeeded so far in localizing the action. Milk of cocaine should always be prepared extemporaneously; as by keeping, the alkaloid is thrown down from the solutions, and much of their efficacy is lost.—*Medical and Surg. Reporter.*

---

THE KEELEY CURE.—Dr. Keeley has taken great pains to keep his remedies a secret, but they have been secured and analyzed by competent chemists, and are now well known. The treatment consists in the use hypodermically four times a day of a solution which shows an analysis of:

| | | |
|---|---|---|
| R | Strychnia sulph. - - | gr. ½. |
| | Atropia, - - - | gr. ¼. |
| | Acid boracic, - - | gr. xv. |
| | Aq. dest, - - - | oz. iv. |

The formula of the tonic taken by the mouth is:

| | | |
|---|---|---|
| R | Ammon muriate, - - | gr. j. |
| | AAloin, - - - | gr. ij. |
| | Tr. cinch comp., - - | oz. iij· |
| | Aq. dest, - - - | oz. j. |

M. S. Teaspoonful every two hours while awake.

During the initial treatment—for the first one, two or three days—much heavier injections of atropia are given, in combination with morphine. Any physician will know at a glance that the quantity of strychnine used is so minute, considering its relative potency, that it can have little effect in modifying the action of the atropia. The tonics support, to a degree, but they, too, have little influence in controlling the symptoms produced by that powerful drug.—*Times and Register.*

SALOPHEIN.—Siebel (*Therap. Monatshefte*, January, '92) says that salol was introduced on account of some of the unpleasant symptoms produced by the salicylates. Salol, however, has not been found harmless, and is contraindicated in cases of renal disease. Salophen is a combination of salicylic acid with acetylparamidophenolether.

It is split up into these two constituents by pancreatic ferment, but not by the acid gastric juice. Any of the drug not so decomposed has been found to be excreted with the fæces. Salophen hinders decomposition when in such quantity as to provide sufficient salicylic acid. It is without taste or smell, and has very feeble toxic properties. At most, symptoms of salicylic acid intoxication would be produced, for the other constituent is without poisonous qualities. Dr. Guttman has spoken favorably of the clinical properties of salophein.—*British Medical Journal.*

---

VALLEY TAN.—With the single exception of the American Indian, it is said there has never existed any people so low in intelligence that they have not devised some means of obtaining alcohol in sufficient strength to produce intoxication. Probably there is no product that is so universal among mankind. Even the inhabitants of the frozen North get alcohol by distilling the products of the arctic fir-trees. It is a singular fact that the American Indian, who never of himself obtained alcohol by any process of distillation, has the most ungovernable appetite for it. There never was a native Indian who would not get drunk if the opportunity offered.

The Mormons of Utah never allow the sale of alcohol among themselves when they are masters of the situation. Yet their religion does not conquer their appetites, for they have an illicit form of it called Valley Tan, which is indigenous to Mormondom. It is said to have all the characteristics of a distillation from sage brush. It looks bitter, smells loud, and tastes yellow, but it gets there just the same.—*Dominion Dent. Journal.*

CLEAN INSTRUMENTS.—Dr. George S. Allen, of New York, in the "International Dental Journal," recommends the use of a one to one thousand solution of bichloride of mercury in rosewater, as an elegant and efficient disinfecting fluid for instruments. Contrary to the common opinion that steel instruments suffer from the use of any solution of the bichloride, he finds that they remain perfectly unaffected after being dipped in it hundreds of times. By the use of rosewater the bug-poison taste of the simple solution is entirely supplanted by an agreeable rose-flavored one. As the plain bichloride decomposes, he advises the preparation of a one per cent. solution from the tartaric sublimate tablets, and the addition of nine parts of rosewater to one of the solution when it is wanted for the disinfection of instruments or for use in the mouth.—*Dominion Dent. Journal.*

---

DR. BARRETT says that a bur is a very dangerous instrument to introduce into a pulp chamber. Where it is necessary to enlarge the opening into a canal, the excavator, he thinks, is the only safe thing, because we know what progress has been made with it. The bur, he contends, buries itself in its own *debris*, so that its exact position cannot be definitely determined.—*Items of Interest.*

---

COLD IN THE HEAD.—For cold in the head while in the acute congestive stage, there is no better remedy than gelsemium. One good large dose, say ten minims of the fluid extract, taken upon going to bed will effectually dispose of this troublesome and uncomfortable affection. One dose is usually sufficient.—*Med. and Surg. Reporter.*

---

A FATAL CURIOSITY.—A weaver in Accrington, England, put a turpentine stupe over his abdomen for the relief of a severe colic. It did not seem to burn as much as it ought, and he struck a match to see what was the reason. The match ignited the turpentine and the man was burned to death.—*Montreal Med. Journal.*

A PROCESS FOR SOLDERING GLASS AND PORCELAIN WITH METALS.—Mr. Cailletet has recently made known to the *Societe de Physique* a process of soldering glass and porcelain with metals. Mechanists, physicists and chemists will appreciate the practical importance of this process, which permits of adapting any metallic object (cock, tube, conducting wire, etc.,) to apparatus in such a way as to prevent any leakage, even under high pressure.

The process is very simple. The portion of the tube that is to be soldered is first covered with a thin layer of platinum. This deposit is obtained by covering the slightly heated glass by means of a brush, with very neutral chloride of platinum, mixed with essential oil of chamomile. The oil is slowly evaporated, and when the white and odoriferous vapors cease to be given off, the temperature is raised to a red heat. The platinum is then reduced and covers the glass tube with' a bright layer of metal. On fixing the tube thus metallized, and placed in a bath of sulphate of copper, to the negative pole of a battery of suitable energy, there is deposited on the platinum a ring of copper, malleable and adhesive.

In this state the glass tube covered with copper can be treated like a genuine metallic tube, and be soldered by means of tin to iron, platinum, and all metals that can be united with tin solder.

The resistance and strength of soldering are very great. Mr. Cailletet has found that a tube of his apparatus for liquefying gases, the upper extremity of which has been closed by means of an ajutage thus soldered, resists pressure of more than 300 atmospheres. The tube, instead of being platinized, may be silverized by raising the glass covered with nitrate of silver up to a heat bordering on red. The silver thus reduced adheres perfectly to the glass, but numerous experiments have caused platinizing to be generally preferred to silverizing.—*La Nature.*

